# Habitat selection by two beluga whale populations in the Chukchi and Beaufort seas

**DOI:** 10.1371/journal.pone.0172755

**Published:** 2017-02-24

**Authors:** Donna D. W. Hauser, Kristin L. Laidre, Harry L. Stern, Sue E. Moore, Robert S. Suydam, Pierre R. Richard

**Affiliations:** 1 School of Aquatic & Fishery Sciences, University of Washington, Seattle, WA, United States of America; 2 Polar Science Center, Applied Physics Laboratory, University of Washington, Seattle, WA, United States of America; 3 Office of Science & Technology, National Marine Fisheries Service, National Oceanic and Atmospheric Administration, 7600 Sand Point Way NE, Seattle, WA, United States of America; 4 North Slope Borough, Department of Wildlife Management, Barrow, AK, United States of America; 5 Freshwater Institute, Fisheries & Oceans Canada, 501 University Crescent, Winnipeg, Canada; Auburn University, UNITED STATES

## Abstract

There has been extensive sea ice loss in the Chukchi and Beaufort seas where two beluga whale (*Delphinapterus leucas*) populations occur between July-November. Our goal was to develop population-specific beluga habitat selection models that quantify relative use of sea ice and bathymetric features related to oceanographic processes, which can provide context to the importance of changing sea ice conditions. We established habitat selection models that incorporated daily sea ice measures (sea ice concentration, proximity to ice edge and dense ice) and bathymetric features (slope, depth, proximity to the continental slope, Barrow Canyon, and shore) to establish quantitative estimates of habitat use for the Eastern Chukchi Sea (‘Chukchi’) and Eastern Beaufort Sea (‘Beaufort’) populations. We applied ‘used v. available’ resource selection functions to locations of 65 whales tagged from 1993–2012, revealing large variations in seasonal habitat selection that were distinct between sex and population groups. Chukchi whales of both sexes were predicted to use areas in close proximity to Barrow Canyon (typically <200 km) as well as the continental slope in summer, although deeper water and denser ice were stronger predictors for males than females. Habitat selection differed more between sexes for Beaufort belugas. Beaufort males selected higher ice concentrations (≥40%) than females (0–40%) in July-August. Proximity to shore (<200 km) strongly predicted summer habitat of Beaufort females, while distance to the ice edge was important for male habitat selection, especially during westward migration in September. Overall, our results indicate that sea ice variables were rarely the primary drivers of beluga summer-fall habitat selection. While diminished sea ice may indirectly affect belugas through changes in the ecosystem, associations with bathymetric features that affect prey availability seemed key to habitat selection during summer and fall. These results provide a benchmark by which to assess future changes in beluga habitat use of the Pacific Arctic.

## Introduction

Arctic marine ecosystems are influenced by many factors but particularly the annual formation and retreat of sea ice, which typically reach seasonal extremes in March and September. Sea ice is a key physical factor affecting the life history and distribution of marine mammals in the Arctic [1,2]. Polar bears (*Ursus maritimus*) and ice-associated pinnipeds such as seals and walruses use sea ice as a platform for foraging, reproduction, and resting (e.g. [3,4]). Seasonal sea ice cycles also indirectly affect access and localized productivity for Arctic marine mammals, which is the primary pathway that sea ice affects the foraging behavior and movements of Arctic cetaceans [5,6].

There are many uncertainties about the effects of recent unprecedented losses of extent, volume, and duration of sea ice [7,8] on Arctic marine mammals, which are also experiencing concurrent increases in anthropogenic pressures [9]. Variability within and among species’ associations and reliance on sea ice further complicates predictions of responses by Arctic marine mammals to loss of ice [10]. Moreover, predictions are complicated by the limited understanding of how most Arctic marine mammal populations select sea ice habitats, especially in conjunction with other habitat features.

Beluga whales (*Delphinapterus leucas*) are ice-associated cetaceans that utilize a broad range of habitats from open water, loose annual pack ice, sea ice edge, and multi-year pack ice [1,11,12]. Nearly 20 genetically-distinct populations have been identified across the Arctic and sub-Arctic. Two of these, the Eastern Chukchi Sea (‘Chukchi’) and Eastern Beaufort Sea (‘Beaufort’) populations, seasonally migrate thousands of kilometers and range to ~80° N into deep areas (>3000 m) with dense pack ice in the Canada Basin [13,14] while also exhibiting philopatry to summering areas [15,16]. Both populations forage on a combination of benthic, epi-benthic, and pelagic prey in shelf and continental slope regions [17,18], and deep diving (>900 m) occurs in basin habitat [19]. Belugas detected by hydrophones and aerial observers during summer-fall in the Alaska Beaufort Sea are associated with the continental slope, are found in open water to heavy ice, and associate with oceanographic features that could enhance foraging opportunities (e.g. a strong Alaska Coastal Current, ACC [11,12,20]). However acoustic studies and aerial surveys cannot differentiate between Chukchi and Beaufort beluga populations, which overlap in distribution in the Alaska Beaufort Sea primarily during September [21]. Sexual segregation occurs in both populations, with adult males occurring farther north and in deeper water [21,22].

Understanding spatial and temporal variability in the use of sea ice and bathymetric habitat between and within these populations can help guide management and conservation, as well as improve predictions about the effects of continued sea ice loss [2]. In this paper, we quantify habitat selection for both sexes of Beaufort and Chukchi beluga whales from July-November. Our primary goal was to use resource selection modeling to quantify population-specific monthly habitat selection, based on location data from 65 belugas tagged with satellite-linked transmitters. We assessed the influence of bathymetry and sea ice on habitat selection within high Arctic summer and fall foraging areas.

## Materials and methods

### Ethics statement

Capture and tagging of Chukchi belugas was conducted under Marine Mammal Protection Act permit nos. 782–1438, and 782–1719 issued to the National Marine Mammal Laboratory (NMML) and number 14610 to the Alaska Department of Fish and Game (ADF&G). Animal care and handling of Chukchi belugas was approved by the Institutional Animal Care and Use Committees of NMML and ADF&G. Tagging of Beaufort belugas was conducted under permits issued by Fisheries and Oceans Canada.

### Study area

**Fig 1:**
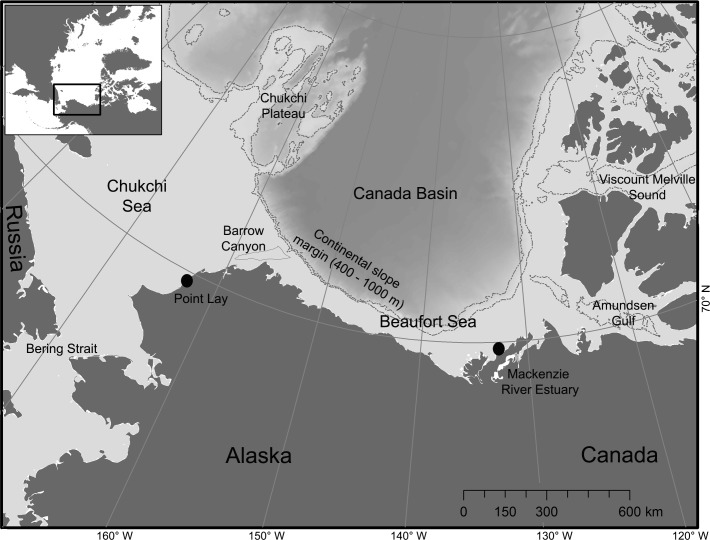
Study area where Chukchi and Beaufort belugas were tagged near Point Lay, Alaska, and the Mackenzie River Estuary, Canada (black circles). Place names and the locations of Barrow Canyon and the 400–1000 m isobaths outlining the continental slope margin are shown.

The Chukchi and Beaufort seas provide a biological and physical connection between the North Pacific and Arctic oceans (Fig 1). The Chukchi Sea is shallow (~50 m mean depth) and broad. The Beaufort Sea is comprised of a narrow continental shelf north of Alaska and western Canada that borders the steep continental slope margins transitioning into the deep (>3,000 m) Canada Basin. The major inflow of Pacific water transits from Bering Strait across the Chukchi Sea and into the Arctic Ocean, bathymetrically channeled via a network of shoals and submarine canyons, including the Barrow Canyon [23]. Advection from Bering Strait primarily flows along coastal northwest Alaska and through Barrow Canyon as the ACC and at times continues along the Beaufort Sea continental slope as a shelfbreak jet with persistent upwelling [24]. These predominant circulation patterns result in an extremely productive region during summer periods of seasonal ice retreat, and the increased duration of the open-water season during the last decade may also contribute to secondary plankton blooms that further enhance regional productivity into the fall [25,26]. Thus, a suite of physical factors related to sea ice and underwater bathymetry influence regional productivity and prey availability that, in turn, presumably impact beluga distribution and habitat selection in the Chukchi and Beaufort seas.

### Beluga capture and monitoring

We used location data from belugas captured and tagged in the Mackenzie River Estuary in July 1993–2005 (Beaufort population) and near the village of Point Lay, Alaska, in late June to early July 1998–2012 (Chukchi population; Fig 1, Table 1). We attached satellite-linked transmitters (Wildlife Computers, Redmond, WA or Sea Mammal Research Unit, University of St. Andrews) to the dorsal ridge of belugas following previously described procedures [13,14,27]. We acquired locations via the Argos satellite system with varying levels of spatial accuracy, so we removed unrealistic locations using the ‘argosfilter’ package in R [28,29] by setting default turn angles and a maximum travel velocity of 6.4 km/h [14]. We then selected the single best quality daily location [21], which could include Argos position qualities 1–3 (error <1.5km to < 250 m), 0 (error > 1.5 km), and A and B (estimated as ~15 and 21 km) [30].

**Table 1:** Number of tagged female and male beluga whales providing locations from the Beaufort and Chukchi populations in July-November, 1993–2012.

					No. locations (No. tagged whales)				
Population	Year	Sex	No. tagged whales	Mean no. days transmitting (range)	Jul	Aug	Sep	Oct	Nov
Beaufort	1993	F	1	27	18 (1)	9 (1)			
		M	3	45 (11–91)	46 (3)	49 (2)	18 (1)	3 (1)	
	1995	F	4	34 (7–68)	53 (4)	30 (3)	6 (1)		
		M	11	37 (23–85)	229 (11)	91 (11)	6 (2)		
	1997	F	3	83 (55–128)	5 (1)	84 (3)	58 (3)	32 (2)	
		M	6	86 (67–120)	11 (6)	177 (6)	149 (6)	82 (6)	11 (1)
	2004	F	3	96 (15–172)	51 (3)	36 (2)	33 (2)	8 (2)	5 (1)
		M	5	182 (17–301)	72 (5)	67 (4)	65 (3)	15 (3)	14 (3)
	2005	F	2	221 (159–283)	45 (2)	60 (2)	60 (2)	62 (2)	16 (2)
	*Total*		*38*	*82 (7–301)*	*530*	*603*	*395*	*202*	*46*
Chukchi	1998	M	5	54 (11–99)	74 (5)	88 (3)	31 (3)	7 (1)	
	1999	F	1	73	14 (1)	28 (1)	10 (1)		
		M	3	75 (56–86)	80 (3)	62 (3)	42 (2)		
	2001	F	3	91 (18–146)	57 (3)	56 (2)	59 (2)	53 (2)	25 (1)
		M	5	75 (16–153)	91 (5)	79 (4)	60 (3)	62 (2)	47 (2)
	2002	F	1	68	12 (1)	16 (1)	7 (1)		
		M	3	64 (46–82)	27 (3)	39 (3)	17 (2)		
	2007	F	2	130 (126–134)	57 (2)	62 (2)	58 (2)	38 (2)	13 (2)
		M	1	521	28 (1)	31 (1)	30 (1)	31 (1)	30 (1)
	2008	M^a^	1		31 (1)	31 (1)	30 (1)	31 (1)	30 (1)
	2010	M	2	133 (101–164)	59 (2)	61 (2)	60 (2)	31 (2)	17 (1)
	2012	F	1	313	22 (1)	31 (1)	30 (1)	31 (1)	31 (1)
	*Total*	* *	*27*	*105 (27–521)*	*552*	*584*	*434*	*284*	*193*

^a^The Chukchi male tagged in 2007 transmitted >18 months, providing locations during July-November 2008 as well as 2007.

### Habitat variables

We established a suite of spatially-explicit environmental variables based on beluga ecology to estimate habitat selection. Specifically, we compiled polar projections of spatial layers for predictors of daily sea ice concentration, seafloor slope, and water depth as well as nearest Euclidean distance to several features: the daily sea ice edge (15% concentration), dense pack ice (90% concentration), shoreline (including barrier islands and mainland), continental slope, and the Barrow Canyon region. We obtained daily sea ice concentration values estimated from satellite passive microwave data (SSM/I), available at a nominal grid resolution of 25 km [31]. A layer of the sea ice edge, defined as 15% sea ice concentration, was created from each daily concentration grid, and we determined the distance of each beluga location to the center of the nearest ice edge pixel. Similarly, we considered the daily distance to dense pack ice (i.e. 90% sea ice concentration). Although whales can navigate through dense pack ice [13,14], we found few of the best quality daily locations positioned in sea ice concentrations >90% (0.6% and 2.5% of Chukchi and Beaufort locations, respectively). The presence of sea ice may impact beluga surfacing behavior and therefore the quality of beluga location data. However, it is likely that the risk of entrapment increases in heavy ice and belugas may avoid dense ice.

Bathymetric features channel major regional currents to link upwelling, local productivity, and ultimately prey availability. We included variables for water depth and percent steepness of the slope as well as proximity to the closest point of continental slope (defined as the 400–1000 m isobaths) and Barrow Canyon features to account for the influence of regional underwater terrain and oceanographic features. We defined a Barrow Canyon feature by selecting the 75 m isobath and clipping it to the seaward boundary (see Fig 1). We extracted depth at each location from the 1 arc-minute resolution ETOPO1 global relief map [32] and calculated percent slope from ETOPO1 water depths using ArcGIS Spatial Analyst tools. We also investigated potential sea ice and ice edge interactions with oceanographic features that could affect beluga distribution (e.g. [12]) by including interaction factors between variables when constructing candidate models.

We controlled for collinearity among habitat variables by calculating variance inflation factors (VIF) with the ‘corvif’ function in the ‘AED’ package in R. Values >3 indicated correlated covariates, which we sequentially removed until all covariate had VIFs <3 [33]. Due to collinearity, the distance to shore predictor variable was eliminated from Chukchi male habitat models.

### Habitat modeling

We examined monthly (July–November) habitat selection for both sexes in each of the two populations, based on previously described differences in monthly distribution and sexual segregation [21,22]. We applied ‘used v. available’ resource selection functions [34] to understand the environmental factors affecting habitat of beluga month-population-sex groups. We established habitat availability for each observed beluga location based on a set of random locations within a circular buffer representing plausible daily movement trajectories. The radius of each buffer was estimated as the 95^th^ percentile of daily displacement rates for each month and population (Table 2). Northrup et al. [35] suggested using >20 random locations to achieve accurate habitat selection estimates, and in our study we selected 25 random locations for each observed position. We used this set of random (i.e. ‘control’) locations to represent habitat availability for each observed (i.e. ‘case’) location. We estimated case-control multivariate conditional logistic regression models to predict the strength of association for habitat variables across summer–fall months, sexes, and populations by applying the ‘clogit’ function in the ‘survival’ package of R that included a robust variance estimator to control for repeated measures of tagged whales.

**Table 2:** Daily 95^th^ percentile displacement rates (km/day) used as buffer distances to generate random ‘control’ locations for Chukchi and Beaufort beluga monthly habitat selection models.

Month	Chukchi	Beaufort
July	116.3	118.6
August	119.4	116.9
September	119.0	127.8
October	119.0	108.2
November	127.3	42.1

We defined a set of 16 candidate models to estimate habitat selection for each month-sex-population group (Table 3). Each model included at least one sea ice variable since we were interested in how seasonal sea ice habitat affected beluga habitat selection. Some candidate models included the variable of sea ice concentration^2^, which has improved model fit for other ice-dependent species by emphasizing variation in dense ice over variation in low ice cover (e.g. [36,37]). We used model selection to identify the most parsimonious set of habitat predictors for each month by estimating Akaike Information Criterion values that were corrected for small sample size (AIC_c_) and using model averaging if multiple models had <2 ΔAIC_c_ [38].

**Table 3:** List of candidate models. Variables include covariates for sea ice concentration (Conc), squared terms of concentration (Conc^2^), proximity to the 15% sea ice edge (Dist_15) and 90% dense pack ice (Dist_90), underwater percent slope (Slope), water depth (Depth), and proximity to the shore (Dist_shore), 400–1000 m continental slope region (Dist_slope), and Barrow Canyon (Dist_canyon).

Model number	Model structure
1	Conc + Conc^2^ + Dist_15 +Dist_90 + Slope + Depth
2	Conc + Slope + (Conc * Slope)
3	Conc + Dist_15 + Dist_90 + Slope + Depth + (Conc*Slope) + (Dist_15 *Slope)
4	Dist_slope + Dist_15 + (Dist_slope*Dist_15)
5	Dist_slope + Dist_canyon + Conc + Conc^2^ + Dist_15 + Dist_90 + Slope + Depth
6	Dist_slope + Dist_canyon + Conc + Dist_15 + Dist_90 + Slope + Depth + (Conc*Slope) + (Dist_15*Slope)
7	Dist_slope + Dist_canyon + Conc + Dist_15 + Dist_90 + Slope
8	Dist_slope + Conc + Conc^2^ + Dist_15 + Slope + Depth
9	Dist_slope + Conc + Dist_15 + Dist_90 + Slope + Depth + (Conc*Slope) + (Dist_15*Slope)
10	Dist_shore + Conc
11	Dist_shore + Conc + Conc^2^
12	Dist_shore + Dist_15 + Dist_slope
13	Dist_shore + Conc + Conc^2^ + (Dist_shore*Conc)
14	Dist_shore + Dist_canyon + Conc + Conc^2^ + Slope
15	Dist_canyon + Conc + Slope + Depth + Dist_90 + (Conc*Slope)
16	Dist_shore + Conc + Slope + Depth + (Conc*Depth)

We applied k-folds cross-validation techniques for case-control habitat selection models to assess the predictive capacity of final selected monthly models for each group [39]. We withheld 20% of our matched observed-random locations to test against the remaining 80% used as a training set for each iteration (k = 5). We calculated the mean Spearman’s Rank correlation to consider the frequency of observed locations for each final selected model, assuming significant correlations were representative of models with high predictive capacity [39,40].

Finally, we mapped predictions of monthly habitat selection for each population using the logistic function to transform coefficients to predicted use [41], scaled for comparison so the maximum prediction was 1.0 [42]. We used monthly composite sea ice layers from the same SSM/I sea ice data [43] for monthly-scale mapping and derived layers for the 15% and 90% sea ice edges from the monthly composite. Based on locations of tagged whales, we defined the spatial extent of monthly (July–November) predicted habitat as the minimum convex polygons (MCPs) describing the entire range of tagged whale locations each month [44]. In addition, we limited spatial predictions to years in which specific sex/population groups were tagged to remain within our scope of inference (see Table 1).

## Results

We acquired 2047 daily locations from Chukchi belugas and 1776 from Beaufort belugas during July–November. Model selection identified a single best model for each month-sex-population combination, except in October for Chukchi males and in November for Beaufort males, when multi-model inference was applied to two top models with <2 ΔAIC_c_ (Table 4). We selected the top model in a few cases where alternatives were within <2 ΔAIC_c_, but were essentially the same models other than a squared ice term or an interaction factor between ice concentration and bathymetry (e.g. models 5 and 7, Table 3). Monthly models were highly predictive of habitat selection, except for Beaufort females in September–November and Beaufort males in October–November when sample sizes of tagged whales were smallest (Table 5).

**Table 4:** Parameters from top resource selection models for female and male Chukchi and Beaufort beluga whales each month (July-November). Bolded values correspond to significant (p<0.05) covariates.

		Chukchi female				Chukchi male				Beaufort female			Beaufort male		
Month	Predictor	Estimate	SE	*p*		Estimate	SE	*p*		Estimate	SE	*p*	Estimate	SE	*p*
*Jul*.	Conc	0.0040	0.0168	0.803		-0.0220	0.0048	**<0.001**		0.0507	0.0165	**0.002**	-0.0100	0.0060	0.096
	Conc^2^	-0.0006	0.0003	**0.028**						-0.0010	0.0003	**0.002**			
	Dist_15					0.0051	0.0014	**<0.001**							
	Dist_90					-0.0006	0.0012	0.637							
	Slope					0.0609	0.0403	0.131					-0.0113	0.0667	0.090
	Depth					-0.0001	0.0001	0.207					0.0005	0.00020	**0.043**
	Dist_slope					0.0030	0.0016	0.057							
	Dist_canyon					-0.0099	0.0015	**<0.001**							
	Dist_shore	-0.0249	-0.0033	**<0.001**						-0.0030	0.0022	0.170	-0.0070	0.0019	**<0.001**
	Conc*Dist_shore	0.0003	0.0001	**<0.001**											
	Conc*Depth												0.0000	0.0000	0.207
*Aug*.	Conc	-0.0283	0.0145	0.051		-0.0007	0.0080	0.932		-0.0257	0.0108	**0.017**	0.0260	0.0110	**0.022**
	Conc^2^												-0.0004	0.0001	**0.004**
	Dist_15	-0.0024	0.0022	0.213		0.0001	0.0012	0.963					0.0045	0.0013	**<0.001**
	Dist_90	0.0001	0.0029	0.749		-0.0014	0.0012	0.241							
	Slope	0.1747	0.0348	**<0.001**		0.1450	0.0242	**<0.001**		0.0669	0.0912	0.463	-0.1885	0.0675	**0.005**
	Depth	-0.0004	0.0002	**0.009**		-0.0003	0.0001	**<0.001**		0.0004	0.0004	0.283	0.0007	0.0001	**<0.001**
	Dist_slope	-0.0100	0.0035	**0.003**		-0.0010	0.0015	0.495					-0.0124	0.0019	**<0.001**
	Dist_canyon	-0.0149	0.0023	**<0.001**		-0.0082	0.0012	**<0.001**							
	Dist_shore									-0.0118	0.0027	**<0.001**			
	Conc*Depth									0.0000	0.0000	0.073			
*Sep*.	Conc	0.0067	0.0106	0.530		-0.0009	0.0066	0.891		0.0049	0.0165	0.765	0.0001	0.0042	0.973
	Conc^2^									-0.0006	0.0003	0.064			
	Dist_15					-0.0035	0.0016	**0.027**					-0.0050	0.0015	**0.001**
	Dist_90	-0.0059	0.0022	**0.008**		-0.0024	0.0016	0.131							
	Slope	0.1831	0.0348	**<0.001**		0.0823	0.0651	0.206					0.0326	0.0613	0.595
	Depth	-0.0004	0.0001	**0.004**		-0.0002	0.0001	0.077					-0.0002	0.0001	0.124
	Dist_slope					-0.0039	0.0019	**0.043**							
	Dist_canyon	-0.0087	0.0020	**<0.001**		-0.0059	0.0016	**<0.001**							
	Dist_shore									-0.0059	0.002	**0.003**			
	Conc*Slope	-0.0144	0.0060	**0.017**		0.0034	0.0012	**0.003**					-0.0027	0.0012	**0.023**
	Dist_15*Slope					0.0002	0.0002	0.281					0.0011	0.0003	**0.002**
	Conc*Dist_shore									0.0001	0.0001	**0.014**			
*Oct*.	Conc	0.0686	0.0187	**<0.001**		0.0045	0.0136	0.739		0.1169	0.0893	0.191	-0.0119	0.0127	0.347
	Conc^2^	-0.0012	0.0000	**<0.001**		-0.0003	0.0002	0.120		-0.0042	0.0035	0.231			
	Dist_15					-0.0057	0.0029	0.051							
	Dist_90					-0.0041	0.0023	0.075							
	Slope	0.1444	0.0480	**0.003**		0.1770	0.0398	**<0.001**							
	Depth					-0.0001	0.0001	0.478							
	Dist_slope					-0.0003	0.0013	0.800							
	Dist_canyon	-0.0041	0.0019	**0.035**		-0.0005	0.0011	0.676							
	Dist_shore	0.0009	0.0020	0.664						0.0002	0.0022	0.941	-0.0032	0.0022	0.143
*Nov*.	Conc	0.0988	0.0274	**<0.001**		0.0547	0.0164	**<0.001**		-0.0072	0.0560	0.897	0.0040	0.0345	0.908
	Conc^2^	-0.0017	0.0005	**<0.001**		-0.0009	0.0002	**<0.001**							
	Dist_15	-0.0121	0.0050	**0.016**		0.0006	0.0026	0.804							
	Dist_90	0.0086	0.0041	**0.038**		0.0001	0.0023	0.951							
	Slope					0.3273	0.0819	**<0.001**					-6.0055	8.7343	0.492
	Depth					-0.0013	0.0005	**0.014**							
	Dist_slope	0.0038	0.0028	0.175		0.0046	0.0020	**0.024**		7.308	4.091	0.074			
	Dist_canyon	-0.0088	0.0034	**0.009**		-0.008	0.0022	**<0.001**							
	Dist_shore												0.0009	0.0067	0.96
	Conc*Slope									-0.3655	0.3381	0.28	-0.0728	0.3414	0.831

**Table 5:** K-folds cross validation results, specifically Spearman’s Rank correlation ($\bar{r_{s}}$) and significance, from top monthly (July-November) habitat selection models for Chukchi and Beaufort male and female belugas.

	Chukchi female		Chukchi male		Beaufort female		Beaufort male	
Month	$\bar{r_{s}}$	*p*	$\bar{r_{s}}$	*p*	$\bar{r_{s}}$	*p*	$\bar{r_{s}}$	*p*
July	0.74	<0.001	0.79	<0.001	0.44	0.035	0.60	0.010
August	0.86	<0.001	0.74	<0.001	0.54	0.013	0.67	<0.001
September	0.62	0.001	0.63	<0.001	0.42	0.091	0.61	0.001
October	0.57	0.004	0.60	0.005	0.21	0.383	0.40	0.059
November	0.68	<0.001	0.70	<0.001	0.23	0.288	0.26	0.21

The best resource selection models indicated that several different factors affected habitat selection for Chukchi and Beaufort belugas (Table 4).

### Chukchi belugas

**Fig 2:**
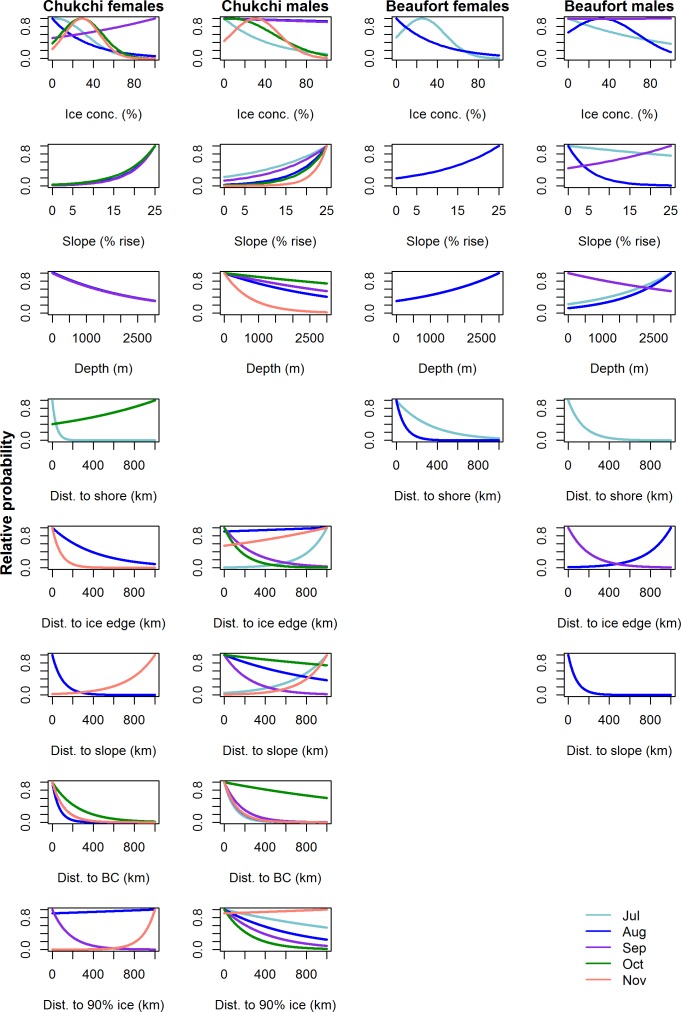
Monthly (July-November) habitat selection predictions, scaled to 1.0, for male and female Chukchi and Beaufort belugas. Missing plots indicate a predictor was not included in the top model, and months with poor predictive capacity for Beaufort belugas are not included (see Table 5).

**Fig 3:**
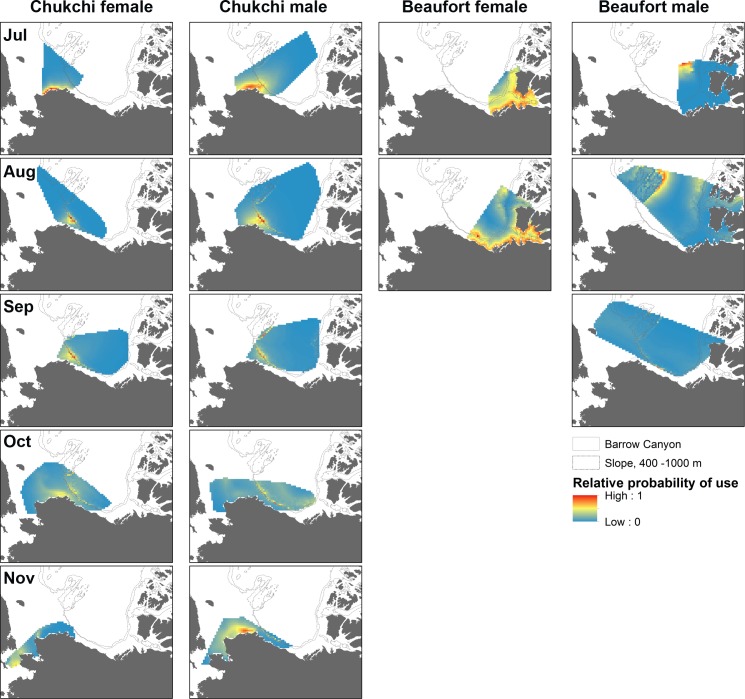
Monthly (July-November, top-bottom) maps of predicted beluga whale use for Chukchi and Beaufort females and males, based on the results of habitat selection models. For each monthly model, predicted habitat use is limited to the scope of inference by averaging across the years when whales were tagged (see Table 1) and restricting spatial extent to the minimum convex polygon of tagged whales in the month. Months with poor predictive capacity for Beaufort belugas are not included (see Table 5).

Sex-specific final models were identified for Chukchi whales in all months except August when the same top model was selected for females and males (Table 4, Fig 2). Proximity to Barrow Canyon and the continental slope regions were included in nearly all monthly models for both sexes, and one of these covariates was often the strongest predictor of habitat selection in each month (Table 4). Sea ice concentration, and sometimes proximity to the ice edge, was a significant predictor in early summer and fall when selected ice concentrations were 20–40% compared to August-September when whales did not select particular ice concentrations and sea ice is typically at its annual minimum extent (Table 4, Fig 2). Percent slope and depth, or an interaction with those variables and ice variables, were also strong predictors of habitat selection for both sexes in several months. Proximity to the coast was the strongest predictor of habitat selection for females in July. These habitat preferences resulted in a high probability of use near Barrow Canyon for Chukchi females from July to October, and for males from July to September and in November (Fig 3). The continental slope regions bordering Canada Basin were also predicted as high use areas for Chukchi males from August to October.

### Beaufort belugas

Final habitat selection models were more distinct between Beaufort males and females than for Chukchi belugas (Table 4, Fig 2). Proximity to Barrow Canyon was not included in top models for Beaufort belugas in any month, nor was the distance to dense ice. For Beaufort females, sea ice concentration was a strong predictor of habitat selection in July and August when predictive capacity was best (Tables 4 and 5). Beaufort females selected ice concentrations <40% in summer (Fig 2), and maps of predicted probability of use reflect a preference for the Mackenzie River Estuary and Amundsen Gulf (Fig 3). Proximity to shore was also a strong predictor from July–September, as well as an interaction of distance to shore with ice concentration in September when Beaufort belugas migrate across the western Beaufort Sea (Table 4). Beaufort males had a strong relationship with deeper water in July and August (Table 4, Figs 2 and 3). Sea ice concentration, and especially proximity to the ice edge, were strong predictors of Beaufort male habitat selection in August when males were predicted to select ~40% ice concentrations, far from the ice edge yet close to slope regions. This included regions such as Viscount Melville Sound where there is a male core area centered over a deep trench [21]. In September, Beaufort males selected areas near the ice edge, although there were also significant interactions between slope and ice concentration as well as distance to the ice edge. There were no significant predictors for either sex in October or November when predictive capacity of the final models was relatively poor (Tables 4 and 5).

## Discussion

Beluga whales, like many migratory marine predators, are confronted by dynamic environmental conditions that influence habitat use over a range of spatial and temporal scales (e.g. [12,45,46]). Habitat selection in Arctic environments can thus indicate important features affecting the distribution of beluga populations, but may also reflect influences of social or sexual resource partitioning [22,47,48]. We developed highly predictive monthly models that revealed large variations in seasonal habitat selection between sex and populations within a remote marine region experiencing rapid environmental change.

### Sea ice selection

Sea ice characteristics were components of our models but rarely as the strongest predictors of monthly beluga habitat use. Sea ice concentration, in particular, typically varies throughout the broad geographic range of these populations. Belugas can navigate heavy ice and are well adapted for inhabiting sea ice, but may also live in ice-free areas for much of the year. We found that belugas from both the Chukchi and Beaufort populations select a range of areas with light (or even ice free) to heavy ice conditions during summer and fall, similar to other reports from the region [11,12].

**Fig 4:**
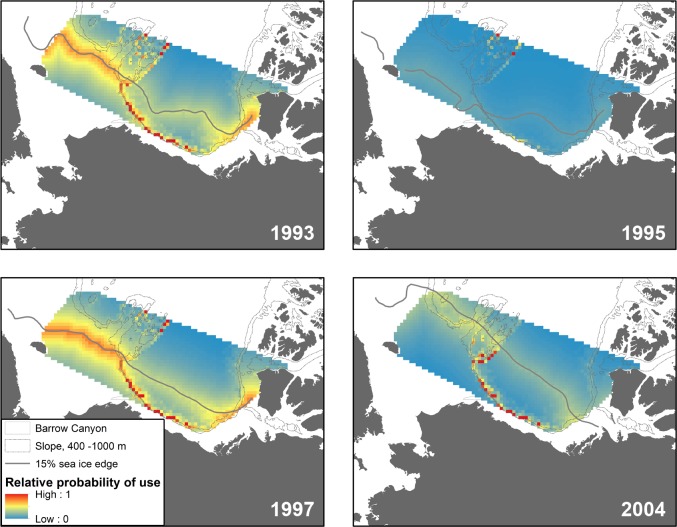
Maps of predicted Beaufort male beluga whale use in September, for years when Beaufort males were tagged (see Table 1). These four years were averaged to produce the map of September Beaufort male relative probability of use in Fig 3. The sea ice edge, derived from monthly ice concentration grids [31], are shown for each year.

Proximity to the sea ice edge was sometimes an important predictor of habitat selection for Chukchi and Beaufort males and Chukchi females. Ice edge habitat has previously been identified as important beluga habitat, including for Beaufort belugas entering the Mackenzie River Estuary in spring [49,50]. During westward migration of Beaufort belugas in September, proximity to the sea ice edge was the strongest predictor for males. However, there has been strong interannual variation in the location of the ice edge in September over the two decades of our study. The predicted habitat map for Beaufort males in September (i.e. Fig 3), averaged across four different years, muted the association with the ice edge in September due to the dynamic nature of ice edge location. Closer examination of individual years revealed the strong selection for the ice edge by Beaufort males in some Septembers (Fig 4). Interaction effects of the ice edge or ice concentration with steep seafloor slope were also strong predictors of Beaufort male habitat selection in September, which resulted in the persistent predictions of high use along the Beaufort Sea slope. Additional information is needed to understand how the continental slope impacts the ice edge in the Beaufort Sea, and in turn how each environmental feature influences beluga habitat selection.

Predation avoidance likely influences habitat selection by belugas. The ice edge could provide a summer refuge from predators, particularly killer whales (*Orcinus orca*) [51,52]. Nearshore and shallow habitat, such as that predicted to affect Beaufort female habitat selection, may also reduce the risk of predation for belugas and the closely-related narwhal (*Monodon monoceros* [51,53]), particularly for females accompanied by calves. Alternatively, coastline or ice edge habitat may serve navigational purposes (e.g. like Arctic terns, *Sterna paradisaea* [54]) or contribute to a suite of navigational cues that help belugas follow a specific course, similar to humpback whales (*Megaptera novaeangliae*) migrating between foraging and breeding grounds [55].

### Oceanographic properties associated with bathymetric features affect population-specific seasonal foraging habitat

Depth, slope, and proximity to bathymetric features (e.g. Barrow Canyon and the continental slope) particularly influenced seasonal habitat selection of belugas. These features guide oceanographic properties including major currents and nutrients, which affect localized productivity and therefore foraging opportunities [24,56,57]. Belugas forage on a combination of epi-benthic and benthic invertebrates and fish [17,18], and foraging dive depths vary among bathymetric regions [19,58]. Fronts in Barrow Canyon and upwelling events along the Beaufort slope can entrain zooplankton and thus attract zooplanktivorous fish that are prey for belugas, such as Arctic cod (*Boreogadus saida* [59]). Localized productivity is further enhanced by advection of Bering Sea zooplankton via Barrow Canyon [60], and Arctic cod abundance is greatest along the continental slope in the Beaufort Sea at depths that coincide with hydrographic fronts (e.g. [61,62]). Chukchi belugas most frequently dive to these depth layers (e.g. 200–400 m) at which Arctic cod are most abundant [19]. These areas coincide with persistently used summer concentration areas for Chukchi belugas [21], as well as a summer ‘hotspots’ for several other marine predators [63,64]. In Viscount Melville Sound (males) and Amundsen Gulf (females), bathymetric features like deep trenches also contributed to habitat predictions for Beaufort belugas [21].

Both beluga populations, especially males, also use the deep (>3000 m) offshore Canada Basin, but the oceanography of this region is relatively poorly known. While our models predicted shelf and slope habitat well, we had fewer beluga data and corresponding oceanographic information for the Canada Basin that could be used to evaluate factors influencing habitat selection there. Belugas can dive >900 m and may focus dives on deeper layers of Atlantic Water origin (200–1000 m) in the Canada Basin [19,58,65], possibly to feed. Our models appeared to underestimate use of the Canada Basin by Chukchi males and females in August and September and overestimate use by Beaufort males in July and August when compared to summer home range estimates that extend into the region [21]. This deep, remote and ice-covered portion of the Arctic Basin is sparsely sampled and its ecology is not well-known. We expect that factors in addition to sea ice and bathymetry affect beluga habitat choice in the region. For example, belugas may track eddies that shed from the slope and potentially entrain prey [66].

Our results suggest Chukchi and Beaufort belugas select habitat with bathymetric features that promote regional productivity and thereby presumably foraging opportunities, but other environmental factors probably also influence habitat selection. In the absence of *in situ* prey sampling, the inclusion of other oceanographic predictors, such as mixed layer depth or eddy tracks, might improve our predictions. However, these features have also not been routinely sampled in the Pacific Arctic. Wind-forcing also impacts hydrography, localized productivity, and sea ice conditions throughout much of the Chukchi and Beaufort seas [67–70], likely impacting beluga foraging opportunities in areas like Barrow Canyon [20] and elsewhere. Beluga habitat models for Cook Inlet, a sub-Arctic Alaskan estuarine system, incorporated information on nearshore river flow and showed an association with mudflats and high flow accumulation that could impact foraging or other behaviors [45].

Similarly, the inclusion of variables on turbidity, substrate, or freshwater flow might also help clarify our results [46], particularly in July when all models predicted close proximity to shore. Molting, which occurs at this time, may occur in fresher and warmer nearshore conditions or be affected by substrate type [71,72]. Freshwater flow from the Mackenzie River peaks in June [73] and may affect the aggregation of Beaufort belugas near the Mackenzie Estuary in spring and early summer [50]. Additional research is needed to better understand whether belugas use nearshore habitats for molting, refuge from predators, as protected waters for calves, or a combination of these factors.

Our interpretation of habitat selection assumes that our sample of tagged whales was representative of each sex and population group in a given month. Our sample sizes were relatively high during the summer but decreased markedly in the fall as tags failed with time (e.g. batteries wane, antennae break, or tags detach). Smaller sample sizes in later months could affect our results in a few ways. First, the relatively poor predictive capacity of our Beaufort beluga fall models (September-November for females and October-November for males) could be a reflection of small samples of whale locations to estimate habitat selection in those months, especially when compared to sample sizes for Chukchi whales (see Table 1). None of the variables we examined were significant predictors of habitat selection of Beaufort belugas in October and November. This was likely related to sample size but could suggest there are other important predictors omitted from our candidate models. Second, our use of MCPs as the area of inference for predictive habitat mapping included all locations used by belugas, and MCPs tend to include unused or rarely-used areas (i.e. [44]). Thus, a single tagged whale with tracks deviating from others could affect what regions are modeled. For example, when a single Beaufort male (i.e., whale 1993–17002) departed the Canada Beaufort Sea ahead of all other Beaufort whales in early August and used the Chukchi Plateau [14], this resulted in a broader August MCP for Beaufort males and affected spatial estimates of relative probability of use.

### Sexual segregation

Distinct morphological or reproductive investment between sexes can result in divergence in the spatial and temporal energetic demands of male and female marine predators (e.g. [74,75]). Belugas are sexually dimorphic; larger males presumably have higher energetic demands than females. Because of their larger size, males can likely dive deeper and longer than females [76]. However, females have high energy demands associated with pregnancy and lactation. Calves wean at about 2 yr and require additional energetic output by nursing females. We found sexual segregation for both beluga populations across summer-fall, although there was generally stronger sexual segregation of habitat predictors for Beaufort than Chukchi belugas. Beluga sexual segregation may be due to divergent energetic and reproductive demands in the summer or a measure to reduce competition. Males were associated with deeper water, heavier ice, and were generally farther from shore than females, as found in earlier analyses of Beaufort beluga data [22,47]. Female belugas closely associate with offspring (both calves and older juveniles), and males likely remain with family groups as juveniles before segregating from females as they mature [77]. Females, especially those accompanied by calves, may choose ice edge or shallow and coastal habitat that reduces predation or risk of ice entrapment. Large adult males are also more physically capable of breaking ice while females with calves would be more vulnerable.

Males likely segregate from females as they mature to exploit alternative prey resources and reduce competition with females and calves, as do other socially-structured cetaceans (e.g. [78]). We found that males selected steeper slopes than females, and proximity to the continental slope was a particularly strong predictor for Chukchi males compared to stronger selection of Barrow Canyon by females. Although there are few regional differences in dive behavior of Chukchi sexes [19,58], fatty acid and mercury analyses of Beaufort belugas indicate that larger, adult males target offshore concentrations of Arctic cod while smaller belugas select more nearshore aggregations [18,79]. Our results add further evidence that males target different prey resources or spatiotemporal concentrations of prey than females. However, age composition of our tagged whales may complicate interpretations of sexual segregation (e.g. [80]) since our analysis combined adult and juvenile whales. Our monthly sample sizes precluded an analysis of age effects, and more data are needed to understand how juveniles may select different habitat than adult males and females.

### Habitat selection in a changing Arctic Ocean

Due to reductions in sea ice over recent decades as well as projections for continued loss [8,81], there is renewed interest in economic development throughout the Arctic [82]. Proposed shipping, tourism, and oil and gas development directly overlap the range of several beluga populations, with potential implications for individual and population-level effects [9]. Our analyses provide quantitative predictions of habitat selection for two beluga populations over the entire open water season when a number of anthropogenic activities are expected to increase. In addition to conservation-related concerns for these marine predators, belugas are integral cultural and subsistence resources for Inupiat and Inuvialuit hunters along the northern and western Alaskan and Canadian coasts [83,84]. Thus, belugas are a sentinel species of primary conservation and cultural value.

Although mitigation of sea ice loss primarily requires global reduction of greenhouse gas emissions, habitat models can help inform management of anthropogenic activities and conservation planning efforts that will increasingly need to identify seasonally important areas for this critical species. Our results suggest that belugas select habitat based on a number of factors. Sea ice variables were rarely the most significant factors affecting beluga summer-fall habitat selection compared with bathymetric features. This suggests perhaps sea ice loss may not impact beluga habitat use. However there are other ways changing sea ice cover could affect belugas, and a recent study showed Chukchi belugas shifted fall migration timing as sea ice freeze-up occurred later in the 2000s [85]. Other environmental changes in the Pacific Arctic are associated with sea ice loss, such as increasing wind and storms that affect primary and secondary productivity [69], which may affect beluga prey. Belugas seem relatively responsive to changing conditions [86], yet further research is needed to clarify the effects of diminishing sea ice and to examine broader long-term impacts for each population. Ultimately, our results provide a benchmark by which to assess future changes in beluga habitat use and help guide regional development of offshore areas.
